# Empowering biologists to decode omics data: the Genekitr R package and web server

**DOI:** 10.1186/s12859-023-05342-9

**Published:** 2023-05-23

**Authors:** Yunze Liu, Gang Li

**Affiliations:** 1 Ministry of Education Frontiers Science Center for Precision Oncology, Faculty of Health Sciences, University of Macau, Macau SAR, China; 2 Cancer Centre, Faculty of Health Sciences, University of Macau, Macau SAR, China; 3 Department of Biomedical Science, Faculty of Health Sciences, University of Macau, Macau SAR, China

**Keywords:** Bioinformatics tool, Web server, Gene set enrichment analysis, Non-programming bioinformatics, Plotting

## Abstract

**Background:**

A variety of high-throughput analyses, such as transcriptome, proteome, and metabolome analysis, have been developed, producing unprecedented amounts of omics data. These studies generate large gene lists, of which the biological significance shall be deeply understood. However, manually interpreting these lists is difficult, especially for non-bioinformatics-savvy scientists.

**Results:**

We developed an R package and a corresponding web server—Genekitr, to assist biologists in exploring large gene sets. Genekitr comprises four modules: gene information retrieval, ID (identifier) conversion, enrichment analysis and publication-ready plotting. Currently, the information retrieval module can retrieve information on up to 23 attributes for genes of 317 organisms. The ID conversion module assists in ID-mapping of genes, probes, proteins, and aliases. The enrichment analysis module organizes 315 gene set libraries in different biological contexts by over-representation analysis and gene set enrichment analysis. The plotting module performs customizable and high-quality illustrations that can be used directly in presentations or publications.

**Conclusions:**

This web server tool will make bioinformatics more accessible to scientists who might not have programming expertise, allowing them to perform bioinformatics tasks without coding.

**Supplementary Information:**

The online version contains supplementary material available at 10.1186/s12859-023-05342-9.

## Background

High-throughput methodologies have revolutionized biomedical research by enabling deep sequencing of genomes, transcriptomes, and epigenomes. These studies generate many gene lists, and interpreting these gene lists can be a significant challenge. Particularly, many laboratories still require the assistance of bioinformaticians for completing fundamental tasks such as retrieving gene information, converting IDs, performing enrichment analysis, and creating plots suitable for publication. However, not all laboratories have an in-house bioinformatician, and most bench scientists lack the skills to use the R programming language. This has resulted in a significant demand for online applications that can perform these tasks. Despite numerous online tools available, many of them failed to meet the needs of bench scientists.

For example, (i) traditional resources used to retrieve gene attributes, including the Entrez Gene from National Center for Biotechnology Information (NCBI) [1], are usually organized in a one-gene-at-a-time format; whereas currently available batch retrieval tools such as The Mouse Genome Informatics Database (MGI) [2, 3], HGNC could only retrieval limited attributes without summaries for gene functions [4, 5]; (ii) most current ID conversion tools, including g:Convert [6] and The Database for Annotation, Visualization and Integrated Discovery (DAVID) [7], are unaware of alias matching, especially when gene symbol and alias are mixed; (iii) the parent-child relationship redundancy of Gene Ontology (GO) terms confounds interpretation [8], increasing the perceived number regarding biologically relevant results; (iv) web servers including WebGestalt [9], Enrichr [10], Web Gene Ontology Annotation Plot (WEGO) [11] and ShinyGO [12] only provides build-in static figure and leave few spaces for users to generate publication-ready illustrations.

To address these issues, we developed an integrated online toolkit called Genekitr. It integrates various functionalities into a single web server, including four modules: GeneInfo module for batch query gene information, IDConvert and ProbeConvert modules for gene and probe identifier conversion, GeneEnrich module for gene enrichment analysis and Plot module for publication-ready plotting. This tool provides a convenient one-stop solution for bench scientists without programming skills.

## Methodology and implementation

### Gene information retrieval module

#### Data collection

Gene information of 317 species, containing 195 vertebrates, 120 plants and 2 bacteria, was retrieved from the quarterly updated Ensembl database (version 108, Oct 2022) [13]. Moreover, NCBI gene annotation for 19 organisms was retrieved by organism-level packages in Bioconductor [14] and UniProt identifiers for 12 organisms were downloaded from UniProt [15, 16], which were subsequently integrated with Ensembl resources as a complement. The gene information mainly includes gene nomenclature, gene function summary, genomic location, gene sequence, gene biotype, and transcript count. Besides, species-specific information was appended. For example, 13,605 human cell marker genes were obtained from the CellMarker database, which assists in identifying and characterizing tissue and cell types [17].

#### Input data

The gene information retrieval module accepts lists of gene identifiers separated by blanks, commas or semicolons. Various types of gene identifiers are accepted, including: Entrez Gene IDs, Ensembl IDs, UniProt IDs, gene symbols and aliases. Gene symbols and aliases are case-insensitive.

#### One-to-many mapping rules

If one-to-many ID mapping occurs, the program performs Boolean operations: firstly, the program will keep records with the maximal number of attributes, then it saves the records with standard chromosome nomenclature instead of unplaced scaffolds and lastly, the program selects the record with the smallest Entrez ID number, as this is usually mapped to a non-predicted genome sequence and is therefore considered official. Besides, the program leaves the result blank if no match is found during this process (see Additional file 1: Fig. S1).

### ID conversion module

The ID conversion module assists in two separate tasks. The first task is ID conversion among gene symbols, gene aliases, Entrez IDs, Ensembl IDs, and UniProt IDs. It is based on the gene information retrieval module and inherits one-to-many mapping rules. The second task is converting human probes to gene symbols or IDs. The human probe annotation data of popular platforms, including Affymetrix, Agilent, Illumina, Phalanx and Codelink, were downloaded from Ensembl by biomaRt [18]. If the probe has no matched gene in the Ensembl database, the NCBI probe annotation data will be loaded from Bioconductor as a supplement. Any unmatched IDs are left as blanks.

### Enrichment analysis module

#### Gene set collection

Gene set raw data files were curated from 11 popular public databases, including 4 libraries of GO (All, biological process (BP), molecular function (MF) and cellular component (CC)), 6 libraries of Kyoto Encyclopedia of Genes and Genomes (KEGG) (Pathway, Module, Enzyme, Network, Drug and Disease) [19], 20 libraries of Medical Subject Headings (MeSH) [20], 22 libraries of Molecular Signatures Database (MsigDB) [21], 256 libraries of Enrichr, and the gene set libraries from WikiPathways [22], Reactome [23], DisGeNET [24], Disease Ontology (DO) [25], Network of Cancer Genes (version 6 and 7) [26] and COVID-19 Gene Set Library [27]. For each database, the term descriptions and gene-term mappings were parsed and retrieved from the raw data files.

#### Enrichment methods

The program supports over-representation analysis (ORA) [28] and Gene set enrichment analysis (GSEA) [29] methods. The ORA method passes a list of gene symbols, gene aliases, Entrez, Ensembl, or UniProt IDs to hypergeometric distribution model, which sampling without replacement:

$$P\left(X \ge k\right) = 1 - \sum_{i = 0}^{k-1}\frac{\left(\genfrac{}{}{0pt}{}{M}{i}\right)\left(\genfrac{}{}{0pt}{}{N-M}{n-i}\right)}{\left(\genfrac{}{}{0pt}{}{N}{n}\right)}$$

where $P$ is the probability of observing $k$ genes in a given gene set, $N$ is the total number of genes in the background set, $M$ is the number of genes within the background set that are annotated to the specific gene set, $n$ is the total size of interested gene list and $k$ is the number of genes within the list which are annotated to the gene set. The GSEA method accepts gene symbols, gene aliases, Entrez or Ensembl IDs with associated fold change values from differential expression analysis. It utilizes fgsea R package to calculate the enrichment scores which represents a gene set is accumulated at the top or bottom of the entire ordered gene list [30]. The nominal p-value is defined as an empirical phenotype-based permutation test.

#### GO term simplifying

15 organism-specific GO term information was extracted from Bioconductor, including *Homo sapiens* (human), *Mus musculus* (mouse), *Rattus norvegicus* (rat), *Drosophila melanogaster* (fruit fly), *Arabidopsis thaliana* (thale cress), *Saccharomyces cerevisiae* (budding yeast), *Danio rerio* (zebrafish)*, Caenorhabditis elegans* (nematode), *Bos taurus* (cow), *Sus scrofa* (pig), *Gallus gallus* (chicken), *Anopheles gambiae* (mosquito), *Canis familiaris* (dog), *Xenopus laevis* (clawed frog) and *Pan troglodytes* (chimpanzee). The relationships between GO terms were retrieved from GO.db [31]. 5 statistical algorithms ("Resnik", "Lin", "Jiang", "Rel" and "Wang") of GOSemSim R package were utilized to calculate semantic similarity for GO BP, CC and MF [32]**.**

### Publication-ready plotting module

Plots are generated based on R packages, including ggplot2 [33], pheatmap [34], VennDiagram [35], ggrepel [36], ComplexUpset [37], ggraph [38], igraph [39].

### Web server implementation

Genekitr web server is implemented on Ubuntu (version 18.04.6) with Shiny R package [40]. Genekitr is accessible from multiple platforms through Microsoft Edge, Chrome, Safari and Firefox.

### Programmatic access

All the functions in Genekitr can be implemented using a local R package called Genekitr, which is available at The Comprehensive R Archive Network (CRAN) repository [41]. Besides, unique features were added to the R package. For instance, the "getPubmed" function helps batch query for PubMed records. "importPanther" function assists in importing and reorganizing GO enrichment analysis results from The Gene Ontology Resource [42], which is powered by PANTHER [43]. "genORA" function supports the comparison of results from multiple gene enrichment analyses.

## Utility and discussion

**Fig. 1:**
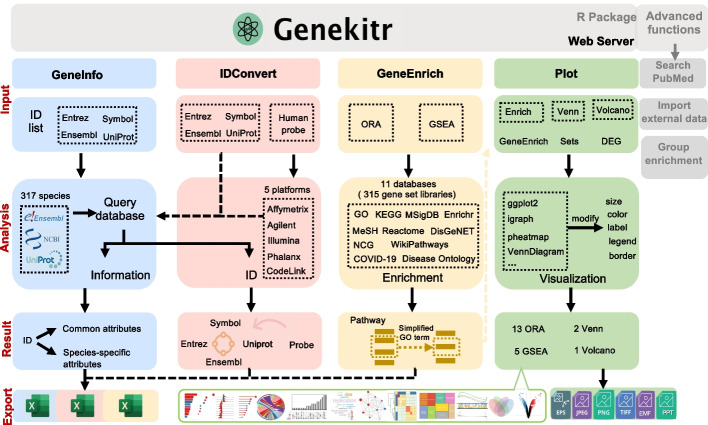
An overview of the functional modules of Genekitr. Genekitr can perform gene information retrieval, identifier (ID) conversion, functional enrichment analysis and online publication-ready plotting. It can plot over-representation analysis, gene set enrichment analysis, Venn diagram, and differentially expressed genes (DEG), in total 21 graph types. The graphs could be customized and exported as files such as Encapsulated PostScript (EPS), Enhanced Metafiles (EMF), Joint Photographic Experts Group (JPEG), Portable Network Graphics (PNG), Tag Image File Format (TIFF), and editable Microsoft PowerPoint (PPT). Both a standalone R package and an online webserver are available to users

Genekitr is an R package and web server that helps biologists analyze large gene sets generated from high-throughput analyses. It comprises four modules to perform gene information retrieval, ID conversion, enrichment analysis, and publication-ready plotting (Fig. 1). Genekitr makes bioinformatics accessible to researchers without programming expertise and enables them to efficiently analyze, present and publish data.

### GeneInfo module

The GeneInfo module allows users to batch-retrieve up to 23 attributes for genes of 317 organisms, including gene symbol, alias, location, biotype, transcript counts, and links to download its sequence and visualize genes in the University of California Santa Cruz (UCSC) genome browser [44]. Importantly, it can batch-retrieve functional summaries of gene products from RefSeq [45]. To help users explore gene information interactively, hyperlinks are provided for databases such as Entrez, Ensembl, HGNC, Online Mendelian Inheritance in Man (OMIM) [46], MGI and International Mouse Phenotyping Consortium (IMPC) [47], which direct users to the official website (Table 1). All retrieved gene information can be downloaded as a Microsoft Excel file, a convenient feature that allows users to analyze the data further. Overall, the GeneInfo module is a valuable tool for exploring gene information and interpreting the potential significance of a list of genes in various biological processes.

**Table 1:** Overview of the hyperlinked data sources

Attribute	Source	Website
EntrezID	Entrez gene	https://www.ncbi.nlm.nih.gov/gene
Ensembl	Ensembl	http://www.ensembl.org/id
UCSC (human and mouse)	UCSC genome browser	https://genome.ucsc.edu/cgi-bin/hgTracks
Sequence (human and mouse)	UCSC sequence	http://genome.ucsc.edu/cgi-bin/das/dsn
Mirbase_ID	MicroRNA database	https://www.mirbase.org
HGNC_ID	HUGO Gene Nomenclature Committee	https://www.genenames.org
OMIM	Online Mendelian Inheritance in Man	https://www.omim.org
MGI_ID	Mouse genome informatics	http://www.informatics.jax.org
IMPC_ID	International Mouse Phenotyping Consortium	https://www.mousephenotype.org

### IDConvert module

**Fig. 2:**
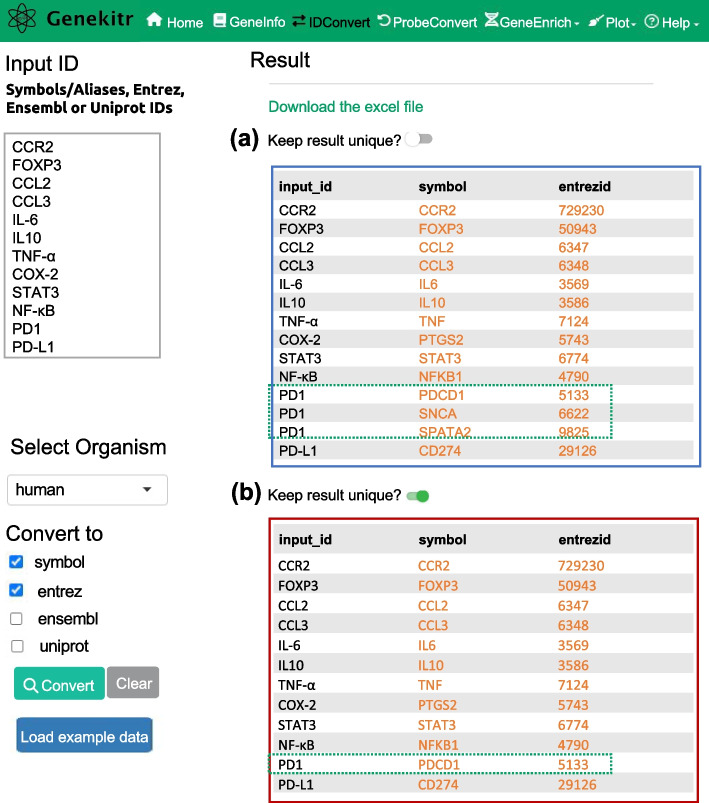
The usage of the gene identifier conversion module in Genekitr. **a** The default behavior of the IDConvert module, which returns all records, including all matches and potential duplicates. **b** The behavior of the IDConvert module when the "unique" button is selected, the module will return only one-to-one mapping results

The IDConvert module in Genekitr enables the conversion of IDs across gene symbols/aliases, Entrez, Ensembl and Uniprot IDs. The results of the conversion also come with hyperlinks that allow users to access additional information. Notably, the module can handle input that includes a mixture of gene symbols and aliases. To assess Genekitr's ability to resolve outdated or unofficial gene symbols, aliases, and identifiers, a gene list related to Shh inhibitors and HH/GLI signaling modulation from a recent publication was analyzed [48]. The gene symbols or aliases were converted to Entrez IDs using Genekitr and five other publicly available tools: DAVID, bioDBnet [49], g:Convert, clusterProfiler [50], and biomaRt. Compared to the other tools, Genekitr was the only one that was able to return Entrez IDs for all searched terms. 4 out of the 5 other tools were able to return Entrez IDs for only 6 out of the 12 queried terms, while bioDBnet was able to recognize gene aliases and return 10 of the 12 terms but could not recognize special characters such as α and κ, in gene names (Table 2). Genekitr also has the ability to provide unique results by adhering to " one-to-many mapping rules". For instance, the human gene known as programmed cell death protein 1 (*PD1*) has three matching symbols: *PDCD1*, *SNCA*, and *SPATA2*. By default, all matching records would be returned, but when the "unique" option is selected, only "*PDCD1*" is returned (Fig. 2). In conclusion, the IDConvert module in Genekitr offers a robust approach to ID conversion by enabling batch queries, handling mixed gene symbols and aliases input, and providing comprehensive results.

**Table 2:** Comparison of gene name converting efficiency*

Searched Terms	Genekitr	DAVID	bioDBnet	g:Convert	clusterProfiler	biomaRt
CCR2	729230	729230	729230	729230	729230	729230
FOXP3	50943	50943	50943	50943	50943	50943
CCL2	6347	6347	6347	6347	6347	6347
CCL3	6348	6348	6348	6348	6348	6348
IL-6	3569	–	3569	–	–	–
IL10	3586	3586	3586	3586	3586	3586
TNF-α	7124	–	–	–	–	–
COX-2	5743	–	5743	–	–	–
STAT3	6774	6774	6774	6774	6774	6774
NF-κB	4790	–	–	–	–	–
PD1	5133	–	5133	–	–	–
PD-L1	29126	–	29126	–	–	–

^*^The table displays Entrez IDs as the output, while the input consists of a mixture of gene symbols and aliases that were converted using the indicated tools

### GeneEnrich module

The GeneEnrich module in Genekitr can perform two types of enrichment analysis: ORA and GSEA. ORA assumes genes operate independently and only considers DEGs based on p-value and fold change; it compares the gene set to a background set and calculates a p-value and fold enrichment to determine significance. GSEA calculates enrichment scores from raw expression levels and detects subtle associations using permutation methods.

**Fig. 3:**
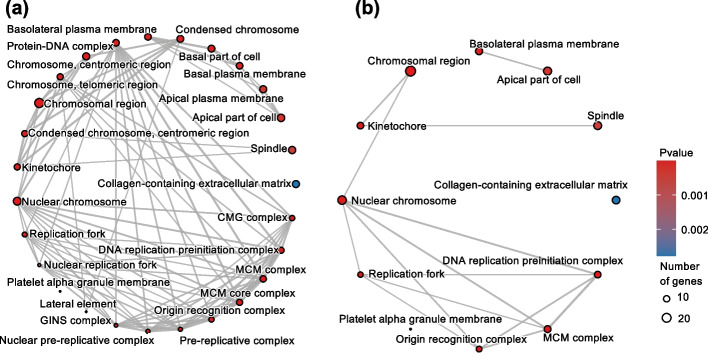
Term network representation of the Gene Ontology (GO) cellular component (CC) enrichment analysis. **a** A circle layout plot that displays all the enriched terms with redundancy. **b** A version of the same diagram with the GO terms simplified

Genekitr's GeneEnrich module incorporates GOSemSim, a GO simplification method, to reduce term redundancy and facilitate more explicit GO enrichment analysis. GO compiles terms of BP, MF and CC as directed acyclic graphs, resulting in a large number of gene sets. However, the parent-child relationship redundancy in the resulting set of GO terms confounds interpretation. To illustrate the effect of simplifying GO terms, we utilized a built-in example generated from differential expression analysis of GSE42872 [51]. GO CC analysis was performed with the option "Simplify GO terms" on or off. All other parameters in the GeneEnrich module are set as default. Both results (see Additional file 2: Table S1, S2) were visualized by the "term network" with "circle" layout in the Plot module (Fig. 3). With the redundancy reduced, researchers could explore GO enrichment analysis more explicitly.

Compared to other web tools for gene enrichment analysis [6, 7, 9, 10, 12, 43, 49, 52–57], Genekitr stands out with several advantages (Table 3). It is the first webserver to integrate the GOSemSim method; it integrates more resources with over 315 libraries covering up to 8213 species; and it has a simple and intuitive interface with a demo file to help users understand the input format. The analysis results can be downloaded in excel format, including comprehensive information such as the "ID" and "Description" of the gene set, "GeneRatio", "BgRatio", "FoldEnrich", "RichFactor" in the ORA method, and "setSize", "normalized enrichmentScore", "geneID" and "geneID_symbol" in the GSEA method (see detail in the online help page of Genekitr). Notably, the gene ID/symbol information can also be used as input for the GeneInfo module of Genekitr to quickly batch-retrieve gene summary information, allowing for a faster and more efficient way to access background knowledge. In addition, Genekitr offers a large number of plotting options for visualization (see below). By using Genekitr itself, researchers can easily generate publication-ready plots.

**Table 3:** Benchmark of Genekitr and existing enrichment analysis webservers

Tool	Method	Gene set libraries	No. of species	No. of plotting types	Customizable plotting	Image type	GO simplification	Availability
WebGestalt [9]	ORA, GSEA, PT^a^	GO, KEGG, + 20 more	12	5	No	PNG, SVG	No	- webserver - R - API
KOBAS [52]	ORA, CGPS^b^	GO, KEGG, + 3 more	- GO: 71—BioCyc: 18 - KEGG: 5944—Reactome: 14 - PANTHER: 41	3	No	PNG	No	- webserver - Python program
g:Profiler [6]	ORA	GO, KEGG, + 7 more	- GO: 821—MIRNA: 16 - KEGG: 255—WikiPathways: 13 - TF: 9 - HP: 414 - CORUM: 3	2	No	PNG	No	- webserver - R - API
DAVID [7]	ORA	GO, KEGG, + 77 more	> 65,000	–	–	–	No	- webserver - API
Gorilla [53]	ORA	GO	8	1	–	PNG	No	webserver
ToppGene [56]	ORA	GO, KEGG, + 135 more	2	1	No	HTML	No	- webserver - API
bioDBnet [49]	ORA	GO, KEGG, + 4 more	6	–	–	–	No	- webserver - API
agriGO [57]	ORA, GSEA	GO	404	2	Yes	PNG, JPEG, GIF, SVG, PDF	No	webserver
Revigo [54]	ORA	GO	25	–	–	–	No	webserver
PANTHER [43]	ORA, GSEA	GO, KEGG	143	5	No	SVG	No	webserver
Enrichr [10]	ORA	GO, KEGG, + 274 more	6	4	No	SVG, PNG, JPG	No	webserver
FunSet [55]	ORA	GO	11	1	No	SVG	No	- webserver - API
ShinyGO [12]	ORA	GO	315	6	Yes	PDF, PNG, SVG	No	webserver
Genekitr	ORA, GSEA	GO, KEGG, + 313 more	- GO: 143—MSigDB: 20 - KEGG: 8213—Enrichr: 5 - Reactome: 11—WikiPathways: 16 - MeSH: 71—Disease specific^c^: 1	- ORA: 13 - GSEA: 5	Yes	EPS, EMF, PPT, PNG, TIFF, JPEG	Yes	- webserver - R

^a^*PT* Pathway topology

^b^*CGPS* Combined Gene set analysis incorporating Prioritization and Sensitivity

^c^The disease specific gene sets for human includes DisGeNET, DO, NCG and COVID-19 Gene Set Library

### Plot module

**Fig. 4:**
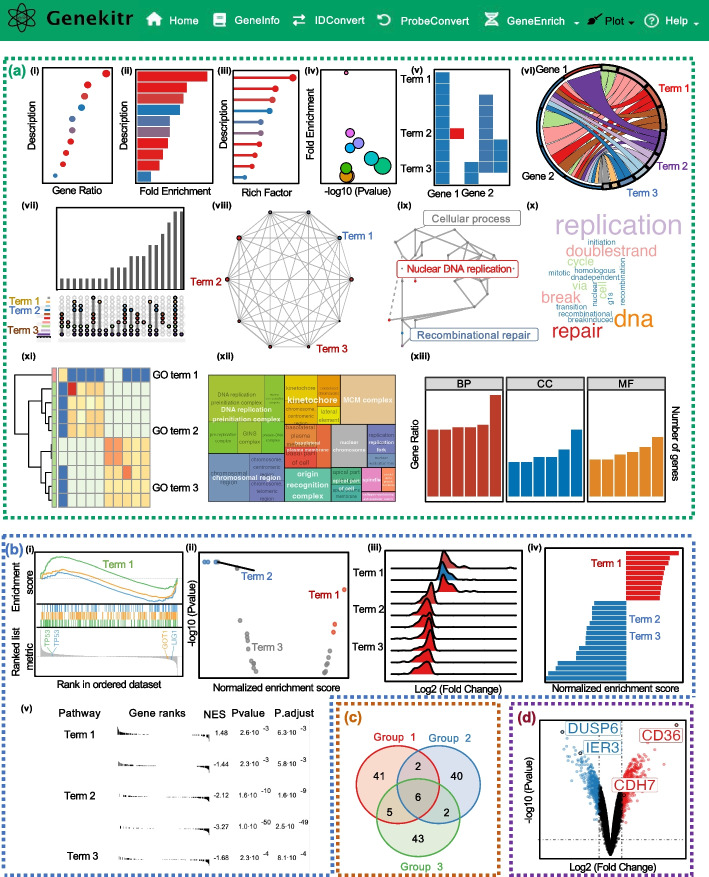
Various types of plots offered by the plot module. **a** 13 plotting types of Over-Representation Analysis (ORA) of gene enrichment, including (i) dot plot, (ii) bar chart, (iii) lollipop plot, (iv) bubble graph, (v) gene-pathway heatmap, (vi) gene-pathway chord graph, (vii) UpsetR interaction plot, (viii) enriched terms network, (ix) enriched term treemap, (x) wordcloud chart, (xi) enriched terms heatmap, (xii) enriched terms tangram and (xiii) WEGO plot **b** 5 plotting types of Gene Set Enrichment Analysis (GSEA), containing (i) classic GSEA plot, (ii) enriched terms volcano plot, (iii) ridge plot, (iv) two-side bar graph and (v) table chart. **c** the Venn plot, which can be used to analyze group interactions. **d** the volcano plot, which can be used in differential gene expression analysis

The plot module offers 21 plot options for tailored data visualization, including 13 options for ORA, 5 for GSEA, 2 for group interactions and 1 for the differentially expressed genes (DEGs) volcano plot (Fig. 4). It has three panels: the upload panel, the parameter panel, and the plot panel.

*Upload panel* Data can be uploaded in either Microsoft Excel spreadsheet (.xlsx), Tab Separated Value (.tsv), or Comma Separated Value (.csv) format. To help clarify the process, a demo file is provided for closer examination, serving as a guide for the required data format and allowing for testing purposes. By clicking the "Upload" button, the data file will be loaded along with preset parameters.

*Parameter Panel* This panel consists of two sections for setting basic and advanced parameters. In the Basic Parameters section, users can select plot types and choose labels for axes, legends, and more. The Advanced Parameters section allows users to customize the plot's color, text size, border thickness, and dot size. It's important to note that the basic parameters vary based on the chosen plot. A key feature in the Basic Parameters section is a drop-down menu, which lists all gene or pathway names from the input file. By selecting one or multiple items, users can directly label their data points on the plot, facilitating the visualization and presentation of their results.

*Plot Panel* By clicking the plot button, the generated plot will be displayed in the Plot Panel with a default resolution of 300 dots per inch (DPI). Users can resize the figure by adjusting the slider bars for width and height. Finally, the figure can be exported in a variety of formats, including Encapsulated PostScript (EPS), Enhanced Metafiles (EMF), editable Microsoft PowerPoint (PPT), Joint Photographic Experts Group (JPEG), Portable Network Graphics (PNG), and Tag Image File Format (TIFF), to satisfy a range of requirements.

The visualization component is crucial in effectively communicating and presenting the analysis results. The plot module offers a range of customization options, including the ability to label data points directly on the plot and export the figures in different sizes and formats, such as EPS, EMF, and editable PPT. These exported figures can be further edited in the related programs, which can be further edited to meet the publisher's requirements. Taken it all, Genekitr offers a comprehensive solution for visualizing, presenting, and publishing the analysis results.

## Conclusions

In summary, Genekitr is a comprehensive toolkit for gene information retrieval, identifier conversion, functional enrichment analysis and plotting. The features of Genekitr include: (i) provision of both a web server and standalone R package, making it accessible to a wide range of users; (ii) the ability to perform batch retrieval of gene summaries and other attributes from up-to-date backend gene databases covering more species; (iii) the ability to handle input that includes a mixture of gene symbols and aliases, resolve outdated gene aliases and provide unique results by adhering to "one-to-many mapping rules" when doing ID conversion; (iv) It supports ORA and GSEA gene enrichment analyses with a simple interface and includes a GO simplification method, and notably, its results provide inputs for batch retrieval of gene summaries for further analysis; (v) Genekitr also enables researchers to easily generate more than 20 types of publication-ready plots with customizability and compatibility with other programs. These features make Genekitr particularly useful for wet-lab biologists with limited bioinformatics expertise who need to conduct basic bioinformatics analysis and generate publication-ready plots.

### Availability and requirements

Project name: Genekitr

Project home page: https://genekitr.org

Operating system(s): Windows

Linux and Mac (web server and R package)

Programming language: R

Other requirements: R 3.6 or higher

License: GPL-3

Any restrictions to use by non-academics: none.

## Supplementary Information

**Additional file 1**. Flowchart of one-to-many mapping rules for gene information retrieval.

**Additional file 2**. 1) GO CC enrichment analysis result without simplification method. 2) GO CC enrichment analysis result after simplification.

## Data Availability

The web server is available at https://genekitr.org. The source code for the web server and the standalone R package is available at https://github.com/GangLiLab/genekitr.

## Abbreviations

BP: Biological process
CC: Cellular component
CRAN: The Comprehensive R Archive Network
DAVID: The Database for Annotation, Visualization and Integrated Discovery
DEGs: Differentially expressed genes
DPI: Dots per inch
DO: Disease Ontology
EMF: Enhanced Metafiles
EPS: Encapsulated PostScript
GO: Gene Ontology
GSEA: Gene set enrichment analysis
HGNC: HUGO Gene Nomenclature Committee
IMPC: International Mouse Phenotyping Consortium
JPEG: Joint Photographic Experts Group
KEGG: Kyoto Encyclopedia of Genes and Genomes
MeSH: Medical Subject Headings
MF: Molecular function
MGI: The Mouse Genome Informatics Database
MSigDB: Molecular Signatures Database
NCBI: National Center for Biotechnology Information
NCG: Network of Cancer Genes
OMIM: Online Mendelian Inheritance in Man
ORA: Over-representation analysis
PNG: Portable Network Graphics
PPT: Microsoft PowerPoint
TIFF: Tag Image File Format
UCSC: University of California Santa Cruz
WEGO: Web Gene Ontology Annotation Plot
