# Supplementary material for: Empowering biologists to decode omics data: the Genekitr R package and web server
Source: BMC Bioinformatics. 2023 May 23;24:214. doi: 10.1186/s12859-023-05342-9 (PMC10205030; doi:10.1186/s12859-023-05342-9)
Supplement: Supplementary file 1 — Additional file 1. Flowchart of one-to-many mapping rules for gene information retrieval. [file 12859_2023_5342_MOESM1_ESM.pdf]

Empowering Biologists to Decode Omics Data: The Genekitr R Package and Web Server

Yunze Liu[1,2,3], Gang Li[1,2,3*]
[1]Ministry of Education Frontiers Science Center for Precision Oncology, Faculty of Health Sciences, University of Macau, Macau SAR, China
[2]Cancer Centre, Faculty of Health Sciences, University of Macau, Macau SAR, China
[3]Department of Biomedical science, Faculty of Health Sciences, University of Macau, Macau SAR, China
*Correspondence: gangli@um.edu.mo

Supplementary Figure and Tables

Table S1. GO CC enrichment analysis result without simplification method.

Table S2. GO CC enrichment analysis result after simplification.

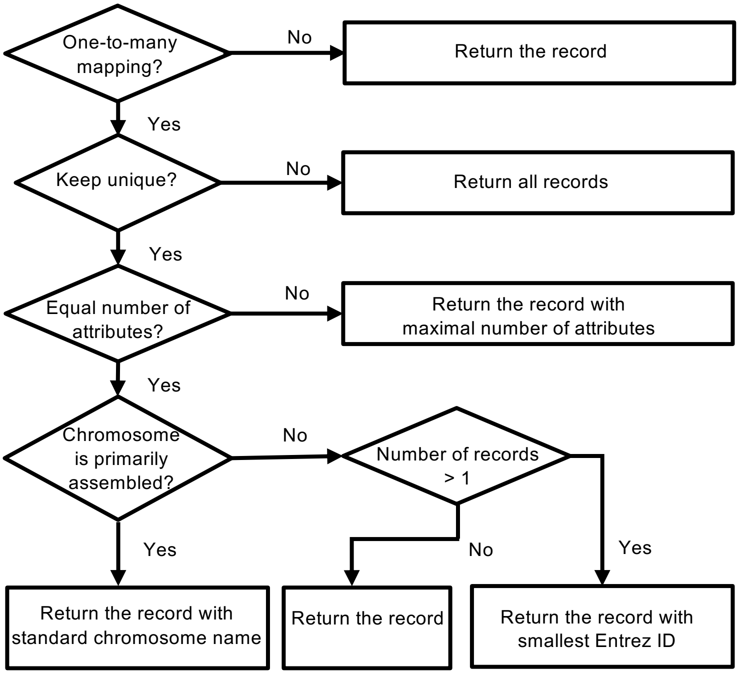

Fig. S1. Flowchart of one-to-many mapping rules for gene information retrieval.
